# Export Control: Post-transcriptional Regulation of the COPII Trafficking Pathway

**DOI:** 10.3389/fcell.2020.618652

**Published:** 2021-01-12

**Authors:** Brittany J. Bisnett, Brett M. Condon, Caitlin H. Lamb, George R. Georgiou, Michael Boyce

**Affiliations:** Department of Biochemistry, Duke University School of Medicine, Durham, NC, United States

**Keywords:** COPII, signaling/signaling pathways, post-translation modification, autophagy, membrane trafficking

## Abstract

The coat protein complex II (COPII) mediates forward trafficking of protein and lipid cargoes from the endoplasmic reticulum. COPII is an ancient and essential pathway in all eukaryotes and COPII dysfunction underlies a range of human diseases. Despite this broad significance, major aspects of COPII trafficking remain incompletely understood. For example, while the biochemical features of COPII vesicle formation are relatively well characterized, much less is known about how the COPII system dynamically adjusts its activity to changing physiologic cues or stresses. Recently, post-transcriptional mechanisms have emerged as a major mode of COPII regulation. Here, we review the current literature on how post-transcriptional events, and especially post-translational modifications, govern the COPII pathway.

## Background and Copii Overview

A complex endomembrane network is a defining feature of eukaryotic cells. Although not connected directly, compartments such as the endoplasmic reticulum (ER), the Golgi apparatus, lysosomes and the plasma membrane exchange materials bidirectionally through vesicles and tubules (Kirchhausen, 2000). Maintaining the protein and lipid compositions of these distinct organelles is essential for their functions and is thus a carefully orchestrated process, critical for cell and tissue physiology. The coat protein complex II (COPII), which mediates anterograde trafficking from the ER, is a highly conserved, key control point for protein sorting (Baker et al., 1988; Ruohola et al., 1988; Barlowe et al., 1994; Routledge et al., 2010; Brandizzi and Barlowe, 2013; Miller and Schekman, 2013; Schlacht and Dacks, 2015).

**FIGURE 1:**
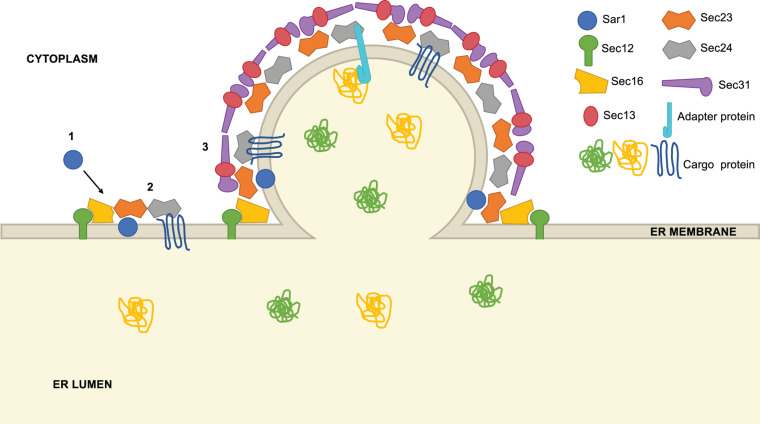
Overview of COPII vesicle formation. COPII vesicle formation proceeds through a series of steps: (1) Sar1 is recruited to the ER membrane at ER exit sites (ERES), marked by Sec16. Using the GEF Sec12, Sar1 exchanges GDP for GTP and inserts an α-helix into the ER membrane, promoting curvature. (2) Sec23 and Sec24 heterodimers are recruited to ERES, binding Sar1 to form the pre-budding complex. Cargo is loaded into the forming vesicle through direct interaction with Sec24, interaction with a Sec24-binding adaptor protein or bulk flow. (3) Lastly, Sec13 and Sec31 heterotetramers assemble around the forming vesicle, promoting further membrane curvature and scission.

The COPII system was discovered and characterized through pioneering studies by Schekman and colleagues beginning 25 years ago (Kaiser and Schekman, 1990; Barlowe et al., 1994; Barlowe, 2020). Our current understanding of the biochemical and structural details of COPII trafficking has been reviewed extensively in several excellent recent articles (Gomez-Navarro and Miller, 2016; Aridor, 2018; Bethune and Wieland, 2018; Brandizzi, 2018; Hutchings and Zanetti, 2019; Peotter et al., 2019). Briefly, COPII vesicle formation begins when the cytosolic GTPase Sar1 binds GTP and inserts an α-terminal N-helix into the ER membrane, a process facilitated by the ER-anchored guanine nucleotide exchange factor (GEF) Sec12 (Figure 1; Gomez-Navarro and Miller, 2016; Aridor, 2018; Bethune and Wieland, 2018; Brandizzi, 2018; Hutchings and Zanetti, 2019; Peotter et al., 2019). Active Sar1-GTP recruits Sec23/Sec24 heterodimers to the ER, which promote Sar1 GTPase activity (Sec23) and mediate carrier loading via direct interactions with cargo and adaptor proteins (Sec24) (Fromme et al., 2008; Routledge et al., 2010; Brandizzi and Barlowe, 2013; Miller and Schekman, 2013; Paczkowski et al., 2015). Then, heterotetramers of Sec13/Sec31 assemble over the Sar1/Sec23/Sec24 pre-budding complex, forming the outer layer of a polyhedral cage that promotes further curvature and Sar1 GTPase-dependent scission (Gomez-Navarro and Miller, 2016; Aridor, 2018; Bethune and Wieland, 2018; Brandizzi, 2018; Hutchings and Zanetti, 2019; Peotter et al., 2019). This complex series of protein and lipid interactions culminates in a mature COPII transport vesicle, typically 60–80 nm in diameter (Gomez-Navarro and Miller, 2016; Aridor, 2018; Bethune and Wieland, 2018; Brandizzi, 2018; Hutchings and Zanetti, 2019; Peotter et al., 2019).

In budding yeast, as well as metazoans, COPII vesicle biogenesis occurs at discrete sites on the transitional ER, called ER exit sites (ERES) (Bannykh et al., 1996; Hammond and Glick, 2000; Bevis et al., 2002; Shindiapina and Barlowe, 2010). ERES are free of ribosomes and marked by Sec16, an ER membrane-associated protein thought to serve as an essential scaffold for COPII assembly *in vivo* (Connerly et al., 2005; Watson et al., 2006). Metazoan ERES are located near the ER-Golgi intermediate compartment (ERGIC) (Schweizer et al., 1990), a cluster of vesicles and tubules containing the mannose-specific cargo receptor ERGIC-53 (Schweizer et al., 1988; Appenzeller et al., 1999), which is distinct from both the ER and Golgi (Schweizer et al., 1991). Unlike COPII vesicles in budding yeast, which fuse directly with the Golgi (Barlowe et al., 1994), mammalian COPII vesicles typically traffic to the ERGIC, and cargoes are subsequently transported to the Golgi by the distinct coat protein I system (Aridor et al., 1995).

The COPII pathway is essential for protein sorting and cell viability in a wide range of organisms (Gomez-Navarro and Miller, 2016; Aridor, 2018; Bethune and Wieland, 2018; Brandizzi, 2018; Hutchings and Zanetti, 2019; Peotter et al., 2019). In humans, genetic defects in COPII impair cargo trafficking and cause a variety of diseases, including skeletal dysplasias, hematologic abnormalities and neurological disorders (Jones et al., 2003; Zhang et al., 2003; Boyadjiev et al., 2006; Lang et al., 2006; Fromme et al., 2007; Schwarz et al., 2009; Merte et al., 2010; Routledge et al., 2010; Wansleeben et al., 2010; Khoriaty et al., 2012; Beetz et al., 2013; Brandizzi and Barlowe, 2013; Miller and Schekman, 2013; Garbes et al., 2015; Moosa et al., 2015; Wang et al., 2020). These examples demonstrate that COPII function is required for tissue and organismal health.

Despite its broad pathophysiological importance and decades of elegant research, significant aspects of COPII trafficking remain obscure. In particular, while the fundamental steps of vesicle assembly are relatively well understood, much less is known about how cells spatiotemporally modulate COPII activity in response to varying cargo sizes, developmental cues, fluctuating signals, metabolic demands or stress (Gomez-Navarro and Miller, 2016; Aridor, 2018; Bethune and Wieland, 2018; Brandizzi, 2018; Hutchings and Zanetti, 2019; Peotter et al., 2019). Flux through the COPII system can change significantly during normal physiological processes, stress and disease states (Harding et al., 1999; Travers et al., 2000; Shaffer et al., 2004; Ron and Walter, 2007; Farhan et al., 2008; Wang and Kaufman, 2012; Hetz et al., 2013; Liu et al., 2019). However, the mechanisms by which COPII responds to these changes are poorly understood, amounting to a significant knowledge gap in the field. There is ample evidence that COPII components are transcriptionally upregulated in response to such cues as differentiation or ER stress (Melville et al., 2011; Izumi et al., 2012; Fang et al., 2015; Ishikawa et al., 2017; Liu et al., 2019), but it is increasingly clear that faster, transcription-independent modes of regulation can also tune COPII activity. Greater knowledge of dynamic COPII regulation will improve our understanding of fundamental eukaryotic cell biology and may reveal new opportunities to treat diseases of aberrant vesicle trafficking in the future. Here, we review the current literature on post-transcriptional regulation of the COPII pathway, with a particular emphasis on post-translational modifications (PTMs) of the coat proteins themselves.

### Sar1

Humans express two paralogous Sar1 proteins, Sar1A and Sar1B, that are ∼90% identical and yet functionally non-redundant *in vivo* (Fromme et al., 2007; Georges et al., 2011). As with other COPII proteins, the distinct functions of ostensibly similar Sar1 paralogs has been puzzling. Tissue-specific expression of each protein is likely part of the explanation. Another, mutually compatible possibility is that Sar1A and Sar1B (and paralogs of other COPII proteins) are differentially regulated by PTMs, affording a greater range of combinatorial control of cargo trafficking. Perhaps consistent with this hypothesis, several studies have reported regulation of Sar1 isoforms by PTMs. (Please see Table 1 for a compilation of the modes of COPII regulation mentioned in this review).

**TABLE 1:** Compendium of COPII relevant post-transcriptional modifications.

Target	Regulation mode	Regulating element	Residue(s)	Proposed function(s)	References
**COPII machinery**					
Sar 1A	miRNA	miR-34C	N/A	Downregulation of Sar1A expression	Bai et al., 2017
	Ubiquitination	Unknown	K166	Unknown	Hornbeck et al., 2015
Sar 1B	Phosphorylation	PKCz	Unknown	Adapting COPII for specialized cargo	Siddiqi and Mansbach, 2012
	Ubiquitination	Unknown	K166	Unknown	Hornbeck et al., 2015
Sec12	N-glycosylation	Unknown	ER lumenal domain	Unknown (protein folding and ER quality control?)	Nakano et al., 1988; d’Enfert et al., 1991; Sato et al., 1996
	O-mannosylation	Unknown	ER lumenal domain	Unknown (protein folding and ER quality control?)	Nakano et al., 1988; d’Enfert et al., 1991; Sato et al., 1996
	Phosphorylation	Hrr25/CK1δ	Unknown	Negative regulation of vesicle budding	Murakami et al., 1999
	Phosphorylation	LTKE	Unknown	Cargo export regulation	Centonze et al., 2019
Sec23	Phosphorylation	Hrr25/CK1δ	T555, S742, T747	Vesicle uncoating and membrane fusion (unidirectionality of trafficking)	Lord et al., 2011
	Ubiquitination	Uba1, Ubc4, Rsp5, Ubp3, Bre5, Cdc48	Unknown	Regulation of Sec23 availability/degradation	Cohen et al., 2003
Sec23A	Phosphorylation	ULK1	S207, S312, T405	Negative regulation of trafficking	Gan et al., 2017
	Ubiquitination	Unknown	C432, C449	Regulation of Sec23A ERES localization (?)	Amodio et al., 2017
	O-GlcNAcylation	O-GlcNAc transferase (OGT)	≥ 26 residues	Collagen trafficking	Cox et al., 2018
	miRNA	miR-21	N/A	Downregulation of Sec23A expression	Li et al., 2016
	miRNA	miR-200	N/A	Downregulation of Sec23A expression	Korpal et al., 2011; Sun et al., 2018
	miRNA	miR-375	N/A	Unknown	Szczyrba et al., 2011; Luo et al., 2013; Bracken et al., 2014; Hart et al., 2014; Eckstein et al., 2019
Sec23B	Phosphorylation	ULK1	S186	Autophagosome biogenesis	Jeong et al., 2018
	miRNA	miR-130a	N/A	Downregulation of Sec23B expression	Ramalho-Carvalho et al., 2017
Sec24	Phosphorylation	Hrr25/CK1δ	Unknown	Unknown	Lord et al., 2011
Sec24C	Phosphorylation	Akt	C-terminal (S888?)	Enhanced Sec23 binding	Sharpe et al., 2011
	Phosphorylation	Unknown (Aurora or PLK?)	S773 and T776 (?)	Suspension of COPII trafficking during mitosis	Dudognon et al., 2004; Kettenbach et al., 2011
	O-GlcNAcylation	OGT	Unknown	Suspension of COPII trafficking during mitosis	Dudognon et al., 2004
	O-GlcNAcylation	OGT	S773, T775, T776	Modulating protein-protein interactions	Cox et al., 2018
Sec24D	Phosphorylation	Akt	Unknown	Enhanced Sec23 binding	Sharpe et al., 2011
	miRNA	miR-605	N/A	Unknown	Lee et al., 2009; Chen et al., 2017
Sec24A	miRNA	miR-101-3p	N/A	Unknown	Su et al., 2009; Liu et al., 2015; Lu et al., 2018
Sec24B	miRNA	miR-576	N/A	Unknown	Coskun et al., 2013;Yarbrough et al., 2014; Liang et al., 2015; Balci et al., 2016
Sec13	Phosphorylation	Unknown	S309	Unknown	Hornbeck et al., 2015
Sec31A	Phosphorylation	PKC?	Unknown	Unknown	Shugrue et al., 1999
	Phosphorylation	Calmodulin-dependent protein kinase II?	Unknown	Unknown	Shugrue et al., 1999
	Phosphorylation	Tyrosine kinases?	Unknown	Unknown	Shugrue et al., 1999
	Phosphorylation	Unknown	S527, S799, S1163, T1165	ER recruitment and trafficking efficiency	Koreishi et al., 2013
	O-GlcNAcylation	OGT	S964?	Subcellular localization and function	Cho and Mook-Jung, 2018
	O-GlcNAcylation	OGT	S451, T658, S666, T674	COPII coat geometry and dimensions?	Cox et al., 2018
Sec 31	Ubiquitination	KLHL12-Cul3-containing E3 ligase	Unknown	Calcium-dependent enlargement of vesicles	Yamasaki et al., 2006; Shibata et al., 2007; Jin et al., 2012; la Cour et al., 2013; McGourty et al., 2016
	Ubiquitination	ARIH1	Unknown	Unknown	Scott et al., 2016
Sec16	Phosphorylation	ERK2	T415	ERES formation/coordination with growth factor signaling	Farhan et al., 2010; Tillmann et al., 2015
	Phosphorylation	ERK7/MAPK15	Residues 1741-1880	Dispersal of Sec16 from ERES	Zacharogianni et al., 2011
	Phosphorylation	ULK1/ULK2	S846	Negative regulation of trafficking	Joo et al., 2016
	Phosphorylation	Unknown	Unknown	Sec23 affinity	Yorimitsu and Sato, 2020
	MARylation	dPARP16	Residues 1805-1848	Negative trafficking response to nutrient deprivation/Sec body formation	Aguilera-Gomez et al., 2016
**Cargoes and receptors**					
SREBP-1c	Phosphorylation	PI3K/Akt	Unknown	Increased affinity for Sec23/24	Yellaturu et al., 2009
Wnt	Palmitoylation	Porcupine	Unknown	Increased Sar1 interaction and COPII trafficking	Sun et al., 2017
TANGO1	Phosphorylation	CK1, PK1	Unknown	Decreased Sec16 affinity/mitotic dissolution of ERES	Maeda et al., 2020
	O-GlcNAcylation	PGANT4	Unknown	TANGO cleavage/loss of secretory granules and apical secretion	Zhang et al., 2014
Sed5	Phosphorylation	PKA	S317	Golgi structural integrity	Weinberger et al., 2005
	Phosphorylation	Unknown	Unknown	Promotion of COPII-dependent protein dissaggregation	Babazadeh et al., 2019

Sar1 regulation by phosphorylation was proposed at least 20 years ago, when it was reported that Sar1 membrane recruitment required not only GTP but also ATP (Aridor and Balch, 2000). Although direct Sar1 phosphorylation was not observed at this time, the kinase inhibitor H89 prevented Sar1 membrane recruitment and the budding of vesicular stomatitis virus glycoprotein (VSVG, a well-characterized COPII model cargo) from microsomes, suggesting kinase regulation of early stage COPII assembly (Aridor and Balch, 2000). Although PKA is a well-known H89 target, the relatively high doses used and the promiscuity of H89 suggested that PKA may not be the relevant kinase (Aridor and Balch, 2000). To investigate this further, the authors also used a PKA peptide inhibitor, and found no inhibition of COPII (Aridor and Balch, 2000). Indeed, other studies confirmed that H89 blocks COPII trafficking at an early biochemical step but indicated that PKA and protein kinase C (PKC) were not the responsible kinases in this case (Lee and Linstedt, 2000). The molecular mechanisms underlying these observations remain unclear.

Later, Sar1B phosphorylation was found to govern the release of pre-chylomicron transport vesicles (PCTV) from the ER (Siddiqi and Mansbach, 2012). Chylomicrons are lipoprotein particles secreted in a COPII- and Sar1B-dependent manner by intestinal enterocytes to transport triglycerides, phospholipids, cholesterol and other cargoes to distant tissues (Siddiqi et al., 2003, 2010). Interestingly, mutations in Sar1B, but not Sar1A, disrupt the secretion of PCTVs and cause chylomicron retention disease in humans (Jones et al., 2003; Georges et al., 2011; Fryer et al., 2014). Although these patients exhibit higher levels of Sar1A, this increase cannot fully compensate for lack of Sar1B (Georges et al., 2011). Isoform-specific regulation of Sar1 may play a role in these unique functions. Siddiqi and Mansbach demonstrated that phosphorylation of Sar1B, but not Sar1A, allowed for the generation of PCTVs *in vitro* (Siddiqi and Mansbach, 2012). The authors showed that fatty acid binding protein 1 (FABP1), which alone can produce PCTVs (Neeli et al., 2007), is sequestered in a cytosolic complex with Sar1B, Sec13 and small VCP/p97-interactive protein (SVIP) (Siddiqi and Mansbach, 2012). Phosphorylation of Sar1B by PKCζ disrupts this complex, freeing FABP1 to bind intestinal membrane and initiate PCTV release (Siddiqi and Mansbach, 2012). Because PCTVs are far larger than typical COPII cargoes, these results may imply a role for Sar1B PTMs in adapting COPII trafficking to specialized physiological functions.

Sar1A has also been identified as the target of miR-34C, a microRNA involved in the development of insulin-producing cells (IPCs) (Bai et al., 2017). Proinsulin secretion from the ER had previously been shown to require Sar1A (Taneja et al., 2009; Fang et al., 2015). More recently, Bai et al. (2017) reported that miR-34C is transiently upregulated to lower Sar1A levels during the differentiation of IPCs from mesenchymal stem cells, but that continued miR-34C expression reduces insulin secretion through downregulation of Sar1A and other targets. Whether these results extend to pancreatic β-cell differentiation *in vivo* remains to be determined.

While studies of Sar1 PTMs have mainly focused on phosphorylation, other modifications have been detected in proteomics screens, such as the ubiquitination of K166 on Sar1A and Sar1B [PhosphositePlus database, www.phosphosite.org (Hornbeck et al., 2015)]. At present, the upstream regulators and downstream functional consequences of these modifications are uncertain.

### Sec12

As the GEF for Sar1, the transmembrane protein Sec12 is essential for COPII vesicle biogenesis *in vivo* (d’Enfert et al., 1991). Early studies of Sec12 revealed that it is both N-glycosylated and O-mannosylated on its ER lumenal domain (Nakano et al., 1988; d’Enfert et al., 1991; Sato et al., 1996). Whether glycosylation impacts the activity of mature Sec12 in COPII trafficking, separate from generic protein folding and ER quality control functions, has not been explored. Sec12 is also phosphorylated by multiple kinases. For example, an early report in yeast indicated that phosphorylation by Hrr25, an ortholog of mammalian CK1δ, negatively regulates COPII vesicle budding at least in part by inhibiting Sec12 function (Murakami et al., 1999). In particular, the growth defects of temperature-sensitive *sec12* mutant strains were partially suppressed by loss-of-function *hrr25* alleles, though the influence of Hrr25 on Sec12 activity in wild type COPII vesicle biogenesis remains unclear (Murakami et al., 1999).

More recently, the Farhan group reported that mammalian Sec12 is a direct target of the leukocyte tyrosine kinase (LTK) (Centonze et al., 2019). Previously, LTK had been identified in two separate functional genetic screens as a regulator of ERES (Farhan et al., 2010; Simpson et al., 2012). Consistent with these observations, the authors showed that LTK phosphorylation of Sec12 regulates the ER export of various COPII cargoes, including mannosidase-II and collagen X (Centonze et al., 2019). Interestingly, LTK also interacts with several COPII cargo receptors, suggesting that it might somehow couple COPII client protein load to Sec12 GEF activity, but this hypothesis remains to be confirmed (Centonze et al., 2019).

### Sec23

**FIGURE 2:**
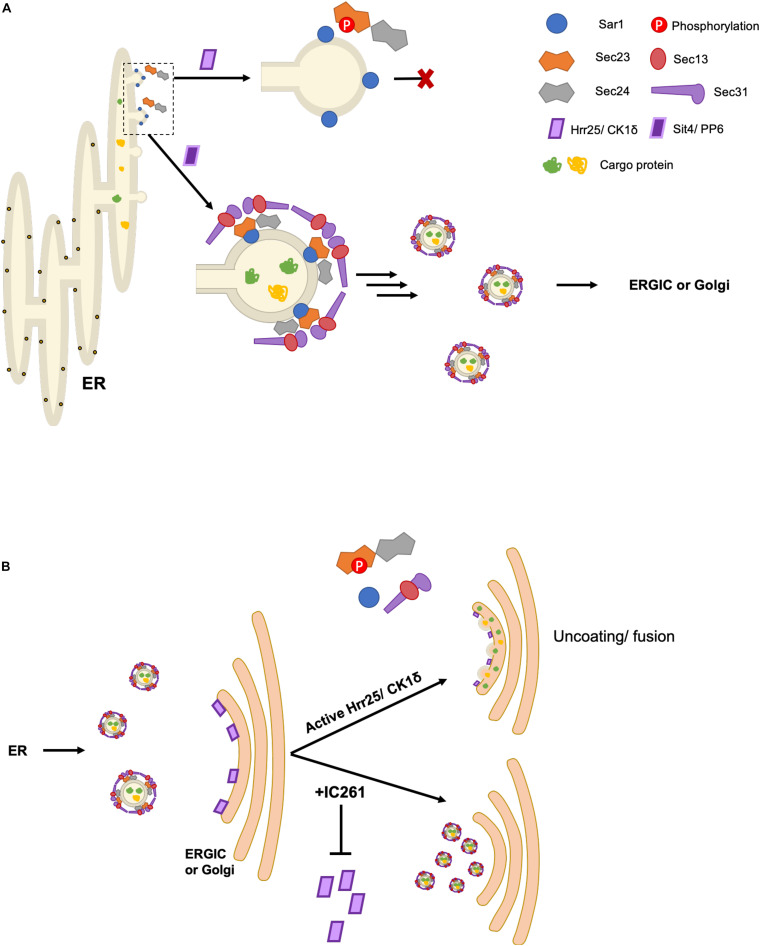
Spatiotemporal control of COPII budding and fusion by Sec23 phosphorylation. **(A)** Phosphorylation of Sec23 by Hrr25 in yeast and CK1δ in mammalian cells prevents interaction with Sar1, inhibiting budding. The phosphatases Sit4/PP6 dephosphorylate Sec23, allowing for COPII vesicle formation. **(B)** Golgi/ERGIC membrane-localized pools of Hrr25/CK1δ phosphorylate Sec23, promoting vesicle uncoating and subsequent fusion with the target membrane. Addition of IC261, an Hrr25/CK1δ inhibitor, prevents COPII uncoating and fusion.

Among all COPII proteins, the post-transcriptional regulation of Sec23 is perhaps the best documented, revealing regulation by a range of mechanisms. For example, the Ferro-Novick group showed that budding yeast Sec23 is phosphorylated on T555, S742, T747 by a Golgi-localized pool of Hrr25, the same kinase that targets Sec12 (Lord et al., 2011). Phosphomimetic mutations at S742 and/or T747 indicated that phosphorylation inhibits Sar1 binding to Sec23, and *in vitro* experiments showed that vesicle budding was blocked by Hrr25 in a kinase activity-dependent manner (Figure 2A; Lord et al., 2011). These data indicate a role for Sec23 *dephosphorylation* in vesicle budding. Conversely, inhibiting Hrr25 via the small molecule IC261 resulted in an accumulation of COPII vesicles docked at the Golgi, suggesting that Hrr25 *phosphorylation* of Sec23 mediates COPII vesicle uncoating and target membrane fusion, but not tethering, *in vivo* (Figure 2B; Lord et al., 2011). The authors also demonstrate similar effects of IC261 treatment on VSVG trafficking in mammalian cells, but whether this occurs through analogous phosphorylation of the paralogs Sec23A and/or Sec23B by CK1δ remains to be demonstrated (Lord et al., 2011). Taken together, these results suggest that spatially and temporally regulated phosphorylation of Sec23 may be a conserved mechanism to ensure the unidirectionality of COPII trafficking, deterring unproductive back-fusion with the ER (Lord et al., 2011).

Phosphorylation has also been implicated in non-canonical functions of Sec23 in the autophagy pathway. Recently, numerous reports have revealed a role for COPII components, including Sec23, in autophagy, likely serving to traffic membrane and perhaps cargo proteins to the nascent autophagosome for macroautophagy or ER-phagy (Graef et al., 2013; Lemus et al., 2016; Jeong et al., 2018; Cui et al., 2019). PTMs are thought to govern this process. For example, the kinase ULK1, a master regulator of autophagy, phosphorylates Sec23A on S207, S312, and T405, inhibiting trafficking from the ER (Gan et al., 2017). S207 is required for Sec23A binding to Sec31 and vesicle assembly, and a phosphomimetic Sec23A S207D mutant reduced interaction with Sec31A and association with the ERGIC marker ERGIC-53, implying that ULK1 blocks outer coat assembly and trafficking (Gan et al., 2017). In a subsequent study, Sec23B, the second mammalian paralog, was reported to be phosphorylated on S186 by ULK1 upon starvation (Jeong et al., 2018). Interestingly, S186 lies within the interaction motif for both Sec24A and Sec24B (two of four mammalian Sec24 paralogs) and the ubiquitin E3 ligase adaptor F-box protein FBXW5 (Jeong et al., 2018). The authors showed that FBXW5 mediates Sec23B ubiquitination and degradation under nutrient-replete conditions (Jeong et al., 2018). However, upon starvation, ULK1 phosphorylation of S186 inhibited FBXW5 binding to Sec23B, thereby reducing Sec23B ubiquitination and stabilizing the protein (Jeong et al., 2018). Furthermore, starvation resulted in the localization of S186-phosphorylated Sec23B-containing vesicles to the ERGIC, where they promoted autophagic flux (Jeong et al., 2018). Based on these results, the authors proposed that ULK1 phosphorylation enlists Sec23B into autophagosome biogenesis (Jeong et al., 2018). In the future, it will be interesting to determine whether coordinated PTMs differentially regulate Sec23A and Sec23B to balance their roles in canonical COPII trafficking and autophagy, and to test whether these mechanisms are conserved across mammalian tissue types, given the varying ratio of Sec23A:Sec23B expression in different organs (Tao et al., 2012; An et al., 2014; Pishesha et al., 2014; Ulirsch et al., 2014; Khoriaty et al., 2018).

Distinct forms of Sec23 ubiquitination are reported to govern core COPII trafficking functions as well. For example, the Dargemont group showed that the E1 enzyme Uba1, the E2 conjugating enzyme Ubc4 and the E3 ligase Rsp5 are required for ubiquitination of Sec23 in yeast, whereas the ubiquitin protease Ubp3 and the adaptor protein Bre5 mediate the removal of this modification (Cohen et al., 2003). The authors demonstrated that monoubiquitinated Sec23 can be either polyubiquitinated and degraded by the proteasome or deubiquitinated, sparing Sec23 function (Cohen et al., 2003). The single modification may regulate the COPII pathway, because biochemical data revealed that Sec23 monoubiquitination reduced its interaction with Sec24 and altered its partitioning between cytosolic and membrane-bound pools, and genetic experiments showed that mutations in *bre5* or *ubp3* caused defects in ER-to-Golgi trafficking (Cohen et al., 2003). In subsequent work, the same group demonstrated that Cdc48, a chaperone-like protein, acts as a Ubp3/Bre5 partner to control the proteasome-mediated degradation of Sec23, underlining the elaborate regulation of Sec23 and COPII activity through this PTM (Ossareh-Nazari et al., 2010). Ubiquitination may also regulate mammalian Sec23. Recently, the Remondelli group reported the unusual monoubiquitination of human Sec23A on two cysteine residues, C432 and C449 (Amodio et al., 2017). These residues may be functionally important for COPII trafficking, as the authors report that a C→A mutation at either site reduces Sec23A occupancy at ERES in cultured mammalian cells, though the mechanisms and consequences of these proposed PTMs remain to be confirmed in trafficking assays (Amodio et al., 2017).

We and others have reported that mammalian Sec23 is also modified by O-linked β-*N*-acetylglucosamine (O-GlcNAc), an essential, intracellular, monosaccharide modification of serine and threonine residues (Teo et al., 2010; Zachara et al., 2011; Cox et al., 2018). Despite the apparent prevalence of Sec23 O-GlcNAcylation, its functional implications long remained unclear. Using mass spectrometry (MS)-based site-mapping, we detected at least 26 O-GlcNAc modifications across multiple domains of human Sec23A (Cox et al., 2018). Sec23A glycosylation is likely dynamic and functionally important, as a small molecule inhibitor of O-GlcNAcase, which removes O-GlcNAc, potentiated Sec23A glycosylation and increased its cytosolic (vs. membrane-bound) localization, compared to vehicle controls (Cox et al., 2018). Moreover, we demonstrated that point-mutations in conserved Sec23A O-GlcNAc sites impaired endogenous collagen transport in human chondrosarcoma cells and failed to fully rescue the collagen trafficking defect and skeletal dysplasia in developing *sec23a* loss-of-function zebrafish (Cox et al., 2018). Together, these results suggest that O-GlcNAcylation is an important mode of vertebrate Sec23A regulation *in vivo*, though the biochemical mechanism through which O-GlcNAc affects Sec23A function has yet to be fully elucidated (Cox et al., 2018).

Finally, many studies have demonstrated that both mammalian Sec23 paralogs can be regulated post-transcriptionally through micro-RNAs, particularly in cancer cell lines and tumors. For example, in colorectal cancer cells, miR-21 downregulates Sec23A protein expression and promotes proliferation, migration and invasion (Li et al., 2016). Sec23A is also downregulated by miR-200 in prostate (Szczyrba et al., 2011; Hart et al., 2014) and breast cancer cells (Luo et al., 2013; Bracken et al., 2014), and may participate in metastasis (Sun et al., 2018). However, the impact of Sec23A regulation by miR-200 may vary by tumor type. In a breast cancer model, miR-200s reduced Sec23A-mediated secretion of metastasis-suppressive proteins, such as insulin-like growth factor binding protein 4 and tubulointerstitial nephritis antigen-like 1, whereas overexpression of miR-200 reduced Sec23A expression, trafficking and metastatic behavior (Korpal et al., 2011). Sec23A expression is also regulated in prostate and breast cancer cells by miR-375 (Szczyrba et al., 2011; Luo et al., 2013; Bracken et al., 2014; Hart et al., 2014; Eckstein et al., 2019). These observations might be functionally important in tumor biology and treatment, because miR-375 overexpression inhibited cell growth and caused apoptosis in prostate cancer cells, but also reduced sensitivity to docetaxel treatment *in vitro* and in *in vivo* xenograft models (Wang et al., 2016). Similarly, in human medullary thyroid cancer, overexpression of miR-375 caused a decrease in cell proliferation and increased sensitivity to the receptor tyrosine kinase inhibitor vandertanib, effects that were attributed to decreased Sec23A expression by siRNA-mediated knockdown experiments (Lassalle et al., 2016). Less is known about the role of micro-RNAs in Sec23B regulation, but a recent study reported that miR-130a suppressed Sec23B mRNA levels in PC3 prostate cancer cells, leading to apoptosis, possibly through the induction of ER stress (Ramalho-Carvalho et al., 2017). In future work, it will be important to determine whether these phenotypic effects of various micro-RNAs are mediated primarily by Sec23-dependent cargo trafficking or autophagy functions in tumors, and to delineate the physiological role (if any) for these micro-RNAs in the COPII pathway in healthy tissue.

### Sec24

Many types of PTMs have been identified on Sec24 proteins, the cargo-selecting subunit of the COPII system, but in most cases the functions of these modifications remain unknown. The Ferro-Novick group reported that Sec24, like Sec23, is phosphorylated by Hrr25 in yeast, but the significance of this observation is not yet clear (Lord et al., 2011). Interestingly, Hrr25 is also found in a complex with the phosphatase Sit4, which itself acts on multiple COPII components, including the Sec24 paralog Lst1, implying a dynamic cycling of phosphorylation on Sec24 (Bhandari et al., 2013). Loss of Sit4 increases the phosphorylation and cytosolic pools of Sec24, Lst1, Sec23, and Sec31 (Roberg et al., 1999; Bhandari et al., 2013). Accordingly, loss of Sit4 or its mammalian ortholog PP6 also delayed the COPII-dependent trafficking of model cargoes, but whether this is mediated by Sec24 phosphorylation in particular, in addition to or instead of the above-mentioned regulation of Sec23, was not clearly defined (Bhandari et al., 2013).

**FIGURE 3:**
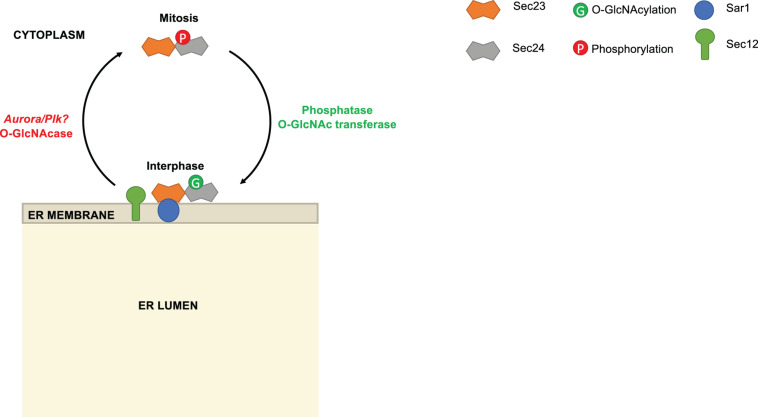
Reciprocal modification of Sec24 by O-GlcNAcylation and phosphorylation. During interphase, Sec24C is O-GlcNAc-modified. As the cell enters mitosis, Sec24C is deglycosylated and phosphorylated. Phosphorylated Sec24C is deficient in membrane association. Phosphoproteomics results suggest that Aurora and/or Polo-like kinase (PLK) may be responsible for phosphorylating Sec24C during mitosis, but this remains speculative.

Some evidence suggests that phosphorylation may regulate Sec24 by modulating its interaction with Sec23 or the ER membrane. In mammalian systems, the serine/threonine kinase Akt was shown to phosphorylate the paralogs Sec24C and Sec24D *in vitro* and *in vivo* (Sharpe et al., 2011). On Sec24C, Akt phosphorylation was localized to the C-terminal 294 amino acids and may reside on S888, which lies in a partial Akt consensus sequence and caused a 40% reduction in phosphorylation when mutated to alanine (Sharpe et al., 2011). Importantly, Sec24C and Sec24D binding to Sec23 was enhanced when cells were treated with insulin-like growth factor-1 (IGF-1) to activate Akt, and this enhancement was suppressed by simultaneous treatment with an Akt inhibitor (Sharpe et al., 2011). These results suggest that Sec24 phosphorylation by Akt or another, Akt-activated kinase may promote COPII trafficking, but this hypothesis has not been rigorously tested in functional assays. Conversely, other phosphorylation events may inhibit Sec24 membrane binding and function. For example, Dudognon et al. (2004) showed that Sec24C is phosphorylated during mitosis (Figure 3). It has long been known that ERES assembly and COPII trafficking are suspended during cell division (Farmaki et al., 1999; Prescott et al., 2001). Intriguingly, Dudognon et al. (2004) showed that phosphorylated Sec24C from mitotic cells could not be recruited to microsomes, indicating that PTMs may modulate COPII traffic through different cell cycle phases. The responsible kinase(s) were not identified, but a subsequent study detected the phosphorylation of Sec24C at S773 and T776 in a proteomic study of Aurora and Polo-like kinase (PLK) signaling, two kinases with crucial roles during mitosis (Kettenbach et al., 2011). It will be interesting to determine in future work whether Aurora, PLK or other kinases are required to suspend COPII trafficking during mitosis through Sec24 phosphorylation.

Intriguingly, Dudognon et al. (2004) also suggested that Sec24 phosphorylation might be regulated reciprocally with O-GlcNAcylation (Figure 3). The authors noted that Sec24C was O-GlcNAc-modified during interphase and deglycosylated during mitosis, when phosphorylation was observed. Many studies have demonstrated that phosphorylation and O-GlcNAcylation compete reciprocally for identical or nearby serine and threonine residues on a wide variety of substrates (Cheng and Hart, 2001; Comer and Hart, 2001; Du et al., 2001; Butkinaree et al., 2010; Wang et al., 2012; Zhong et al., 2015; Leney et al., 2017). Recently, we have extended these observations in the case of Sec24C. Our MS analysis of purified human Sec24C revealed O-GlcNAc sites on S773, T775, and T776 (Cox et al., 2018). According to crystal structures of Sec24C, these residues lie at its juxtamembrane surface, suggesting that bulky glycosylation at these sites could alter membrane binding (Mancias and Goldberg, 2008). Moreover, as noted above, S773 and T776 are reported to be phosphorylated by Aurora, Plk or another kinase (Kettenbach et al., 2011), leading to the intriguing hypothesis that reciprocal, site-specific O-GlcNAcylation and phosphorylation might regulate recruitment of Sec24C to the ER (Figure 3). Additionally, O-GlcNAcylation of Sec24 and other COPII components may mediate their protein-protein interactions. We treated human cells with a photocrosslinking GlcNAc analog and observed the crosslinking of Sec24B, Sec24C, and Sec23A to as-yet unidentified partner proteins, indicating that O-GlcNAc residues on COPII proteins can directly contact other interactors (Cox et al., 2018). Experiments to determine the functional effects of Sec24 O-GlcNAcylation are underway.

Like other COPII proteins, Sec24 expression is regulated post-transcriptionally by microRNAs. Sec24A is a target of miR-101-3p (Lu et al., 2018), which suppresses invasion and metastasis in multiple cancers, including hepatocellular carcinoma (Su et al., 2009), adenoid cystic carcinomas (Liu et al., 2015), and gastric cancer (Lu et al., 2018). Sec24D is a target of miR-605, a micro-RNA found only in primates (Lee et al., 2009). Like miR-101-3p, miR-605 suppresses tumor growth in a melanoma model (Chen et al., 2017). Finally, miR-576 is encoded within an intron of the *sec24b* gene, is induced during viral infections and is thought to inhibit excess inflammation by targeting STING (stimulator of interferon genes), MAVS (mitochondrial antiviral-signaling protein), and TRAF3 (TNF receptor-associated factor 3) (Yarbrough et al., 2014). miR-576 is also down-regulated in non-melanoma skin cancer (Balci et al., 2016), early T-cell precursor acute lymphoblastic leukemia (Coskun et al., 2013), and bladder cancer (Liang et al., 2015). Overexpression of miR-576 in bladder cancer significantly reduced proliferation, although this may be due partly to targeting of cyclin D1, rather than Sec24 (Liang et al., 2015). As with Sec23, it will be interesting to determine the physiological functions of Sec24-targeting mi-RNAs in healthy cells, as well as in tumors.

### Sec13

The outer coat protein Sec13 is the only core COPII component with a single homolog in mammalian cells. Although it is likely to be subject to dynamic regulation, almost nothing is known about PTMs of Sec13. Public repositories [PhosphositePlus, (Hornbeck et al., 2015)] indicate that Sec13 phosphorylation at S309 has been detected in nearly a dozen separate datasets from high-throughput phosphoproteomics experiments, but these observations have not been validated through independent methods and the functional significance of Sec13 phosphorylation remains unexplored.

### Sec31

Experiments in yeast, protists and mammalian cells indicate that the outer coat protein Sec31 is regulated by phosphorylation, ubiquitination, and O-GlcNAcylation, but the functional consequences remain incompletely understood in most cases (Salama et al., 1997; Jin et al., 2012; Koreishi et al., 2013; Hu et al., 2016; McGourty et al., 2016; Cho and Mook-Jung, 2018). While large-scale MS studies have detected phosphorylation on more than 30 sites on human Sec31A (one of two paralogs) (Olsen et al., 2006; Dephoure et al., 2008; Hornbeck et al., 2015), only four of those sites (S527, S799, S1163, T1165) have been independently verified by a biochemical approach (Koreishi et al., 2013). Similarly, many reported sites of ubiquitination still await validation (Lumpkin et al., 2017; Akimov et al., 2018).

Sec31 PTMs were discovered when the protein itself was first identified in yeast, when it was shown to be phosphorylated via ATP (γ^–32^P) labeling and immunoprecipitation (IP) (Salama et al., 1997). The functional significance of phosphorylation was supported by the observation that alkaline phosphatase treatment of Sec31 reduced vesicle budding by 50% in an *in vitro* assay (Salama et al., 1997). When mammalian Sec31 was discovered, potential phosphorylation sites for PKC, calmodulin-dependent protein kinase II and tyrosine kinases were noted, but no experiments were performed to confirm modification (Shugrue et al., 1999). Later, Koreishi et al. (2013) analyzed Sec31A purified from human cells and identified four phosphorylated residues (S527, S799, S1163, T1165). Sec31A with S/T→A mutations at all four sites (4A) localized more strongly to ER membranes and co-IP-ed more efficiently with Sec23 than did wild type Sec31, suggesting a functional impact of phosphorylation on efficient ER recruitment (Koreishi et al., 2013). Importantly, the 4A mutant also demonstrated defective trafficking of COPII cargoes, further supporting a functional effect of phosphorylation (Koreishi et al., 2013). Residual Sec31 phosphorylation was detected on the 4A mutant, however, suggesting that other regulatory phosphorylation sites might remain to be discovered (Koreishi et al., 2013). Regulation of Sec31 by phosphorylation also appears to be broadly conserved across evolution. Hu et al. mutated seven putative phosphorylation sites on the *Trypanosoma brucei* Sec31 ortholog and found that this 7A mutant caused growth defects that phenocopied an RNAi knockdown of the gene (Hu et al., 2016). Trafficking of a model COPII cargo was blocked by Sec31 knockdown but could be rescued by re-expression of wild type Sec31 or a phosphomimetic mutant at all seven sites (7D), but not with the 7A mutant (Hu et al., 2016). Collectively, these studies indicate that phosphorylation functionally regulates Sec31, but future studies would benefit from determining the impact of phosphorylation at individual sites.

Evidence of Sec31 monoubiquitination arose from the discovery of an interaction between Sec31 and Kelch-like family member 12 (KLHL12), a substrate adaptor for Cullin-3 (Cul3)-containing ubiquitin ligase complexes (Jin et al., 2012). Jin et al. (2012) observed *in vitro* binding and monoubiquitination of Sec31 by KLHL12-Cul3, and subsequently confirmed these interactions in cells, as dominant-negative Cul3 or genetic knockdown of KLHL12 strongly diminished Sec31 ubiquitination. Similarly, Scott et al. (2016) reported that Sec31 may also be ubiquitinated by Ariadne RBR E3 ubiquitin protein ligase 1 (ARIH1), because ARIH1 knockdown in mammalian cells also reduced Sec31 ubiquitination. Polyubiquitination of Sec31 has not been observed, and proteasome inhibition does not impact Sec31 levels, suggesting mono-, not poly-, ubiquitination of Sec31 is regulatory (Jin et al., 2012; Scott et al., 2016). Interestingly, overexpression of KLHL12 stimulated COPII vesicle enlargement from 70 nm up to 500 nm in diameter and revealed that these carriers contained collagen, a large COPII cargo (Jin et al., 2012). Overexpression of a KLHL12 mutant that cannot bind Sec31 or a Cul3 mutant that blocks Sec31 ubiquitination did not cause vesicle enlargement, and knockdown of KLHL12 or Cul3 resulted in ER retention of collagen in various mammalian cell models (Jin et al., 2012). These data suggest that monoubiquitination of Sec31 by KLHL12-Cul3 stimulates the enlargement of COPII vesicles required for collagen trafficking (Jin et al., 2012). This model is complemented by work investigating ubiquitin-specific protease 8 (USP8), which deubiquitinates Sec31 in mammalian cells (Kawaguchi et al., 2018). Sec31 and USP8 co-IP-ed, and siRNA knockdown of USP8 increased Sec31 ubiquitination and stimulated collagen secretion (Kawaguchi et al., 2018). Importantly, large COPII vesicles induced by KLHL12 overexpression were suppressed by USP8 overexpression, resulting in collagen retention in the ER (Kawaguchi et al., 2018). Together, these studies provide evidence that the export of collagen and perhaps other large cargoes depends on the reversible monoubiquitination of Sec31. In the future, it will be important to confirm the functional role of the endogenous KLHL12, Cul3, and USP8 machinery in collagen trafficking *in vivo*.

Some evidence suggests the monoubiquitination of Sec31 may respond to calcium signaling (McGourty et al., 2016). In mammalian cells, Sec31 co-IPs and co-localizes at ERES with the calcium binding protein α-1,3/1,6-mannosyltransferase (ALG-2) in a calcium-dependent manner (Yamasaki et al., 2006; Shibata et al., 2007; la Cour et al., 2013; McGourty et al., 2016). In an *in vitro* COPII vesicle formation assay, ALG-2 reduced budding in the presence of calcium in a dose-dependent fashion (la Cour et al., 2013). Thus, calcium-stimulated ALG-2 binding of Sec31 may delay COPII vesicle budding, providing the time needed for vesicles to enlarge sufficiently to encapsulate massive cargoes, such as collagen (la Cour et al., 2013). In addition to binding Sec31, ALG-2 also co-IP-ed with KLHL12, suggesting that ALG-2 might modulate the ubiquitination of Sec31 by Cul3-containing E3 ligase complexes (McGourty et al., 2016). Consistent with this notion, a rise in cytoplasmic calcium levels stimulated Sec31 monoubiquitination and potentiated the formation of enlarged, collagen-containing COPII vesicles, but not when ALG-2 was siRNA-depleted (McGourty et al., 2016). In the absence of ALG-2, no enlarged COPII vesicles were observed, and collagen accumulated in the ER (McGourty et al., 2016). In contrast, Yamasaki et al. (2006) observed that the trafficking of VSVG, a small cargo, is not affected by ALG-2 depletion. These data support the notion that COPII trafficking of massive cargoes may require calcium signaling and Sec31 monoubiquitination for cage enlargement, but that this is dispensable for smaller cargoes.

We and others have also found that Sec31A is O-GlcNAcylated (Wells et al., 2002; Dudognon et al., 2004; Zachara et al., 2004; Cox et al., 2018), though the functional impact of this modification is as-yet unclear. Recently, Cho et al. reported that Sec31 O-GlcNAcylation on S964 regulates its subcellular localization and function (Cho and Mook-Jung, 2018). However, the authors did not provide direct evidence of O-GlcNAcylation at any site (e.g., by MS) and instead relied on indirect pulldowns with wheat-germ-agglutinin, a low-affinity GlcNAc-binding lectin (Cho and Mook-Jung, 2018). Therefore, the proposed O-GlcNAcylation of S964 remains unverified. In separate work, we used MS site-mapping to identify four specific sites of O-GlcNAcylation on Sec31A, S451, T658, S666, and T674, all of which reside in the α-solenoid domain (Cox et al., 2018). This region of Sec31A is known to mediate its protein-protein interactions in the outer coat lattice and is thought to form a flexible hinge, allowing for coat expansion to accommodate collagen and other large cargoes (Stagg et al., 2006, 2008; Fath et al., 2007; Hutchings and Zanetti, 2019). Therefore, it is tempting to speculate that Sec31A O-GlcNAcylation, like monoubiquitination, may influence COPII coat geometry and dimensions. Experiments are underway to delineate the functional effects of site-specific O-GlcNAc modification of human Sec31.

### Sec16

**FIGURE 4:**
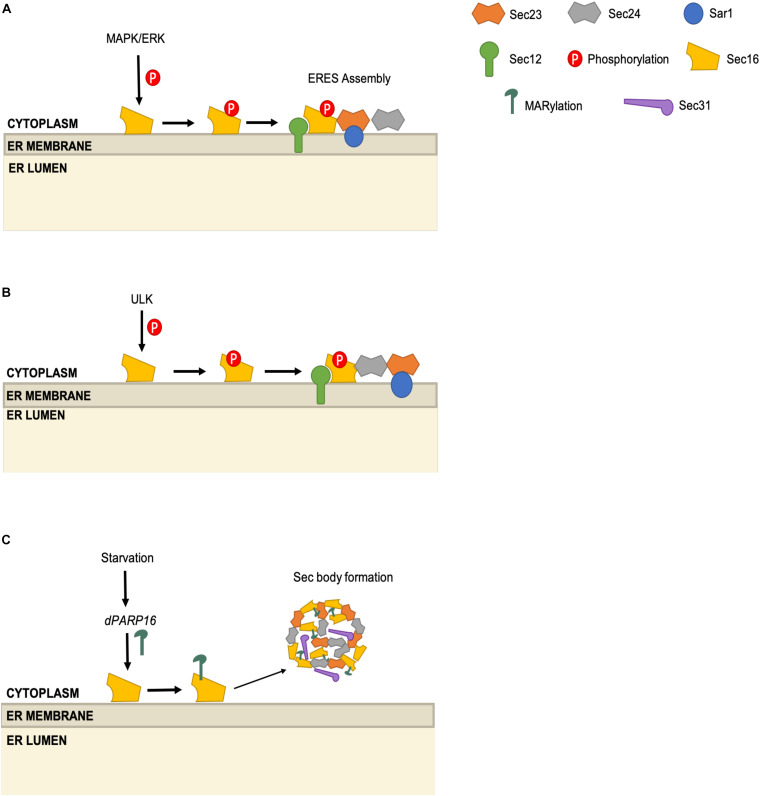
Multi-modal regulation of the ERES scaffolding protein Sec16. **(A)** ERK2 phosphorylates Sec16 on T415 and promotes ERES assembly. **(B)** ULK1 and ULK2 phosphorylate Sec16A and Sec16B. Sec16 phosphorylation promotes Sec16-Sec24 interaction. **(C)** Upon starvation, dPARP16 promotes MARylation of Sec16, which leads to the formation of Sec bodies, composed of Sec16, Sec23, Sec24, and Sec31.

Sec16 is a large, evolutionarily conserved, membrane-associated protein that interacts with several COPII components at ERES. Although dispensable for *in vitro* vesicle formation, Sec16 is thought to facilitate COPII carrier assembly *in vivo* and is required for cell viability (Espenshade et al., 1995; Supek et al., 2002; Watson et al., 2006). As might be expected from this essential role, Sec16 is also subject to several types of post-transcriptional control. In an early example, Farhan et al. (2010) found evidence of Sec16 phosphorylation in a functional genetics screening for siRNAs that perturbed the trafficking of an ERGIC-53 model cargo in HeLa cells. Pursuing hits in the mitogen-active kinase (MAPK) cascade, the authors showed that ERK2 knockdown reduced the number of ERES and trafficking of the COPII client α1-antitrypsin by approximately one-third (Farhan et al., 2010). Conversely, *in vitro* assays revealed that ERK2 potentiated vesicle budding, implicating an early step in the COPII pathway (Farhan et al., 2010). Subsequent biochemical experiments revealed that ERK2 phosphorylates Sec16 on T415, and an unphosphorylatable T415I mutant supported less ERES assembly than did wild type Sec16 (Figure 4A; Farhan et al., 2010). ERK2 phosphorylation of Sec16 may connect growth factor signaling to COPII activity, because expression of active Ras kinase increased Sec16 phosphorylation and ERES number, and later work showed that mitogen stimulation requires Sec16 T415 in order to increase ERES number as well (Farhan et al., 2010; Tillmann et al., 2015).

Remarkably, a second functional genetics screen suggested a role for Sec16 in MAPK signaling in a separate system. Zacharogianni et al. (2011) used an RNAi screen in *Drosophila* cells to identify kinases required for the proper trafficking of Fringe, a COPII client protein. In this case, the activity of ERK7 or its human homolog, MAPK15, was shown to promote dispersal of Sec16 from ERES (Zacharogianni et al., 2011). Serum starvation produced similar effects on Sec16 localization, and this effect was ERK7-dependent as well (Zacharogianni et al., 2011). Interestingly, the authors showed that T415, the ERK2 phosphorylation site mentioned above, is not needed for these ERK7-driven effects on Sec16, and instead amino acids 1741-1880 of Sec16 were required (Zacharogianni et al., 2011). Although positive evidence of Sec16 phosphorylation by ERK7 (e.g., MS data) is lacking, these results nevertheless indicate a complex role for Sec16 in linking growth factor and MAPK signaling to COPII trafficking (Zacharogianni et al., 2011).

Sec16 phosphorylation may also connect the COPII pathway to the autophagy machinery. Using an MS proteomics approach, the Kundu group demonstrated that the autophagy kinases ULK1 and ULK2 interact directly with and phosphorylate Sec16A and Sec16B (two mammalian paralogs) (Joo et al., 2016). Intriguingly, the authors showed that the ULK1/2-Sec16 interaction impacts COPII trafficking, rather than autophagy, as ULK-deficient murine cells or *C. elegans* exhibit lower ERES numbers, reduced Sec16-Sec24C colocalization and decreased trafficking of serotonin transporter (SERT), an obligate Sec24C client cargo (Joo et al., 2016). The authors identified S846 as the crucial ULK phosphorylation site on Sec16, demonstrating that the unphosphorylatable mutant S846A supported fewer ERES and less SERT trafficking, as compared to wild type or an S846D phosphomimetic mutant allele (Figure 4B; Joo et al., 2016). Given the above-mentioned role of ULK in directing Sec23 function away from canonical COPII trafficking and toward autophagosome formation (Gan et al., 2017; Jeong et al., 2018), it will be important for future studies to determine how and when ULK-mediated Sec16 and Sec23 phosphorylation are integrated to balance the COPII and autophagy pathways.

As a counterpoint to these results, a recent study by the Sato lab indicated that Sec16 phosphorylation may play little or no role in controlling ER transport or autophagy in budding yeast, suggesting a possible evolutionary divergence in this mode of COPII regulation (Yorimitsu and Sato, 2020). The authors showed that deletion of the N-terminal 565 amino acids of Sec16 led to accumulation of COPII client cargoes in the ER and a marked decrease in autophagy (Yorimitsu and Sato, 2020). However, unphosphorylatable Sec16 mutants, in which either the N-terminal, C-terminal or all expected Sec16 phosphorylation sites were mutated to alanine, showed no change in ER export or autophagy, relative to wild type (Yorimitsu and Sato, 2020). Interestingly, the authors observed that when all Sec16 phosphorylation sites were mutated to alanine, Sec16 showed a nearly twofold increase in interaction with Sec23 (Yorimitsu and Sato, 2020). This finding is reminiscent of the Kundu lab’s observation in metazoans that Sec16 phosphorylation by ULK affects its ability to interact with the Sec23/24 complex, specifically modulating transport of Sec24C client cargoes (Joo et al., 2016).

Sec16 activity may also be regulated by mono-ADP-ribosylation (MARylation), a reversible PTM that influences a wide range of biological processes (Krishnakumar and Kraus, 2010; Fujimori et al., 2012; Hu et al., 2013; Watanabe et al., 2016; Cohen and Chang, 2018). Aguilera-Gomez et al. (2016) developed fluorescent probes to monitor MARylation in *Drosophila* cells and observed that this modification was induced in response to amino acid starvation. Previous work had suggested that amino acid starvation induced the sequestration of COPII proteins into “Sec bodies” in *Drosophila* cells, thought to be a way to suspend trafficking during nutrient deprivation while maintaining the COPII machinery in reserve (Amodio et al., 2009; Zacharogianni et al., 2014). However, the mechanisms governing Sec body formation remained largely unknown. Aguilera-Gomez et al. (2016) found that the ribosyltransferase dPARP16 is required for both starvation-induced MARylation and Sec body formation, suggesting a direct regulatory role (Figure 4C). The authors observed MARylation signal near ERES, and found that C-terminal residues 1805–1848 of Sec16 (but not certain other COPII proteins, such as Sec23) were required for this signal and could induce Sec body formation when overexpressed in the absence of stress (Aguilera-Gomez et al., 2016). These results suggest that Sec16 may receive nutrient cues through MARylation to tune COPII activity. It will be interesting to determine whether Sec16 is directly MARylated in fly and mammalian cells, and to define the mechanism that connects amino acid sensing to ribosyltransferase activity.

## Cargoes, Cargo Receptors, and Accessory Proteins

**FIGURE 5:**
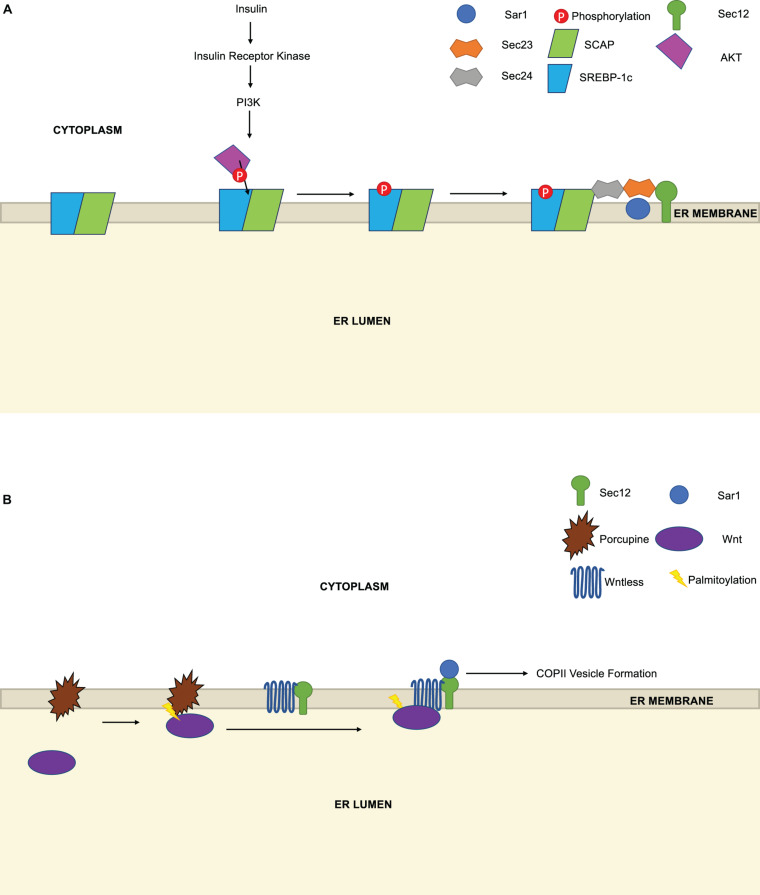
Regulation of COPII trafficking by cargo PTMs. **(A)** Insulin stimulation, which activates the PI3K pathway, induces the phosphorylation of sterol regulatory element binding proteins (SREBP) by Akt. Phosphorylation increases the affinity of the SREBP-1c/SREBP cleavage-activating protein (SCAP) complex for Sec23/Sec24 and promotes COPII-dependent trafficking to the Golgi. **(B)** The acyltransferase Porcupine is necessary for the palmitoylation of Wnt, which enhances its binding to preformed Wntless/Sec12 complexes and facilitates Wnt secretion by COPII.

One under-explored question in the COPII field is whether the export of specific cargoes is regulated by modification of the cargoes themselves, their receptors or accessory proteins, as opposed to changes on the core COPII machinery. A few examples indicate that this mode of regulation may be more widespread than is currently appreciated. For instance, Yellaturu et al. (2009) reported that phosphorylation of the COPII cargoes sterol regulatory element binding proteins (SREBPs) regulates their interaction with COPII components (Figure 5A). SREBPs are transmembrane transcription factors that govern the expression of genes controlling cholesterol homeostasis and *de novo* fatty acid biosynthesis, with SREBP-1c as the major isoform in liver and fat (Shimano and Sato, 2017; Brown et al., 2018; DeBose-Boyd and Ye, 2018). Previously, insulin stimulation was known to induce SREBP-1c mRNA and protein in hepatocytes, but whether it affected trafficking or other post-translational aspects of SREBP-1c function was not fully clear (Hegarty et al., 2005). Yellaturu et al. (2009) used a Sar1-glutathione S-transferase pulldown assay and an *in vitro* microsome system to discover that insulin stimulation induces the phosphorylation of SREBP-1c at the ER (Figure 5A). This, in turn, enhances the affinity of the SREBP-1c cleavage-activating protein (SCAP)-SREBP-1c complex for Sec23/Sec24, and its subsequent trafficking to the Golgi for site-1 and site-2 protease processing, requisite steps in SREBP signaling (Yellaturu et al., 2009). This phosphorylation event is likely functionally important because alkaline phosphatase treatment of membranes from hepatoma cells abolished the insulin-induced enhancement of SREBP-1c/SCAP binding to Sec23/Sec24 (Yellaturu et al., 2009). The phosphoinositide 3-kinase (PI3K)-Akt pathway, which is induced by insulin, was required for these effects on trafficking, as chemical or genetic inhibition of PI3K/Akt blocked insulin-induced SREBP-1c phosphorylation in the hepatoma system (Yellaturu et al., 2009). Finally, the authors demonstrated that Akt can phosphorylate SREBP-1c *in vitro* (Yellaturu et al., 2009), suggesting that the PI3K/Akt pathway may govern COPII trafficking at both the level of Sec24 phosphorylation, noted above (Sharpe et al., 2011), and cargo phosphorylation. These results may also explain prior observations that the PI3K inhibitor LY294002 disrupts COPII-dependent transport of SCAP, while activation of PI3K/Akt by insulin-life growth factor-1 treatment increases SCAP transport (Du et al., 2006).

A second example of trafficking control through cargo PTMs is provided by Sun et al. (2017) in their study of Wnts (Figure 5B). Wnts are an important class of cell surface receptors that are required for embryonic development in animals and are dysregulated in human cancers (Steinhart and Angers, 2018; Patel et al., 2019). Wnt secretion requires the transmembrane protein Wntless, which binds Wnts directly, but the mechanisms controlling Wnt/Wntless vesicle assembly and trafficking are incompletely understood. Here, the authors used an IP/MS approach to show that Wntless binds to Sec12 and several other COPII proteins (Sun et al., 2017). Interestingly, they further demonstrated that the Wntless/Sec12 interaction promotes Wnt secretion, and PTMs are required for this effect (Sun et al., 2017). Wnts are known to be acylated as part of their maturation (Steinhart and Angers, 2018; Patel et al., 2019). Sun et al. (2017) showed that palmitoylation of Wnts promotes the Wntless/Sec12 interaction, thereby enhancing the interaction (presumably indirect) between Wntless and Sar1, and potentiating subsequent COPII-mediated trafficking. This signaling is likely required for optimal Wnt secretion, as an inhibitor of the acyltransferase Porcupine prevented Wnt palmitoylation and reduced the Wnt/Wntless/Sar1 interactions, although the Wntless/Sec12 interaction persisted (Sun et al., 2017). The authors propose that Wnt maturation (including palmitoylation) and subsequent binding to pre-formed Wntless/Sec12 complexes facilitate Sar1 recruitment and COPII vesicle assembly and export (Sun et al., 2017). In this model, Wnt palmitoylation could be understood as a licensing PTM, serving as a prerequisite for a properly liganded cargo to be efficiently loaded into and secreted by COPII carriers.

Another interesting example is provided by transport and Golgi organization 1 (TANGO1), an ERES-localized transmembrane protein that acts as a cargo receptor for collagen (Bard et al., 2006; Saito et al., 2009; Raote et al., 2018). TANGO1 recruits ERGIC membranes to ERES in order to generate the carrier sizes necessary for collagen and other large cargo (Bard et al., 2006; Saito et al., 2009; Raote et al., 2018). TANGO1 has also been shown to form rings around ERES (Raote et al., 2017) and interact with another ER-membrane resident protein, cutaneous T-cell lymphoma-associated antigen 5 (cTAGE5), to localize Sec12 and Sec23 to ERES as well (Saito et al., 2009, 2011, 2014). The case for its importance in ERES formation is further strengthened by a study indicating that TANGO1 recruits and coordinates with Sec16 to properly organize ERES (Maeda et al., 2017). Moreover, PTMs of TANGO1 have been implicated in modulating its role as an ERES assembler and organizer. Recently, Maeda et al. demonstrated that TANGO1 is a substrate of the kinase CK1 and the phosphatase PP1, and that TANGO1 phosphorylation by CK1 reduces its interaction with Sec16, leading to a mitosis-linked dissolution of ERES (Maeda et al., 2020). Another study in *Drosophila* showed that loss of PGANT4, an O-GalNAc transferase, resulted in TANGO1 cleavage and subsequent loss of secretory granules and apical secretion (Zhang et al., 2014). However, more work is needed to identify the specific residues modified by these PTMs. Indeed, many TANGO1 phosphorylation sites have been reported, but the regulatory roles of these remain to be explored (Chen et al., 2009; Grimsrud et al., 2012; Wilson-Grady et al., 2013; Mertins et al., 2014; Parker et al., 2015; Pinto et al., 2015; Minard et al., 2016; Sacco et al., 2016; Williams et al., 2016; Robles et al., 2017; Degryse et al., 2018).

In a final example, phosphorylation of the transmembrane v-SNARE machinery, which is responsible for COPII vesicle fusion with its target membrane, has profound effects on endomembrane structure within *S. cerevisiae* (Weinberger et al., 2005). Studies have focused on Sed5, an essential SNARE that interacts with several other SNAREs in distinct complexes to mediate COPII vesicle-ERGIC fusion and COPI-dependent retrograde fusion (Holthuis et al., 1998; Mossessova et al., 2003; Weinberger et al., 2005). Weinberger and colleagues demonstrated that aspartate substitution of S317 of Sed5, a PKA phosphorylation site, resulted in elongation of the ER, accumulation of vesicles in the cytoplasm, aberrant retrograde trafficking, and growth defects (Weinberger et al., 2005). Conversely, substitution of S317 with alanine resulted in a stacked, ordered Golgi atypical of *S. cerevisiae*, but no defects in endomembrane function (Weinberger et al., 2005). The authors suggest a model in which phosphorylation of Sed5 at S317 leads to dispersal of Golgi membrane, whereas dephosphorylation leads to mammalian-like Golgi stacks (Weinberger et al., 2005). More recent work has also implicated Sed5 PTMs in COPII-dependent protein quality control (Babazadeh et al., 2019). Researchers observed that phosphorylation of Sed5 in *S. cerevisiae* led to an increase in COPII association with heat-shock protein 104 and an increase in ER-to-Golgi trafficking, promoting protein disaggregation (Babazadeh et al., 2019). It will be interesting to determine whether phosphorylation of syntaxin-5, the mammalian ortholog of Sed5, exerts similar effects on trafficking or organelle morphology.

## Unknown Targets—New Biology Awaiting Discovery

As the examples above illustrate, PTMs regulate many, if not all, components of the COPII pathway. A range of other studies also implicates PTM signaling in COPII trafficking, but without identifying the relevant targets, highlighting the considerable amount of interesting biology that remains to be characterized. Indeed, as noted previously, functional genetic screens showed that dozens of kinases and phosphatases are required for normal COPII trafficking (Farhan et al., 2010; Simpson et al., 2012). The discovery of Sec12 as an important target of LTK, which emerged as a hit in both screens (Farhan et al., 2010; Simpson et al., 2012), demonstrates the promise of functional genomics methods to reveal new aspects of COPII regulation.

Directed experiments with chemical inhibitors have also implicated PTM signaling in COPII trafficking. In an early example, Pryde et al. (1998) discovered a link between serine/threonine phosphatases and vesicle assembly. It has long been known that the Golgi fragments in regulated fashion during mitosis in order to partition to daughter cells (Nelson, 2000; Valente and Colanzi, 2015). This process involves the suspension of trafficking pathways, but how that is accomplished remains incompletely understood. Because the PP1/PP2A inhibitor okadaic acid (OA) also causes reversible Golgi fragmentation, Pryde et al. (1998) used it as a tool to investigate the mechanisms behind this phenomenon. The authors showed that OA treatment arrests the trafficking of a model COPII cargo, and that ERGIC-53 co-fractionated with Sec13 in control cells but accumulated in the rough ER fraction, away from Sec13, in OA-treated cells (Pryde et al., 1998). Sec13 was not displaced from the membrane by OA treatment, indicating that the inhibitor may act by blocking cargo entry into COPII vesicles (Pryde et al., 1998). Unfortunately, no specific phosphoprotein substrate of PP1/PP2A was implicated in these observations.

In a similar vein, Wang and Lucocq (2007) used a semi-permeabilized cell system and the microtubule poison nocodazole, which arrests cells in mitosis by disrupting the spindle, to examine the regulated suspension of COPII trafficking. Consistent with Pryde et al. (1998), the authors found that a cocktail of phosphatase inhibitors prevented COPII cargo loading into vesicles (Wang and Lucocq, 2007). They then tested candidate kinases to determine which may account for this effect and found that inhibition or immunodepletion of p38 MAPK restored trafficking even in the presence of nocodazole (Wang and Lucocq, 2007). Conversely, nocodazole treatment activated p38, supporting the specificity of these results, although no p38 substrates were identified to explain the apparent effects on COPII function (Wang and Lucocq, 2007). In addition, the authors were unable to detect p38 activation in the same cell system using a mitotic shake-off method, suggesting that p38’s role in COPII regulation may be due to microtubule disruption, rather than a natural feature of mitosis, at least in this case (Wang and Lucocq, 2007).

Chemical kinase inhibitors have also provided tantalizing clues to as-yet uncharacterized COPII regulation. As described above, H89, an ATP-competitive isoquinolinesulfonamide, has been a useful tool in several contexts (Aridor and Balch, 2000; Lee and Linstedt, 2000). In another example, Jamora et al. (1999) used H89 to inhibit PKCμ in rat kidney epithelial NRK cells, observing that it prevented Golgi fragmentation induced by Gβγ, a heterotrimeric G protein that may interact with PKCμ. VSVG reporter assays confirmed that H89 arrests COPII-dependent trafficking from the ER in this system (Jamora et al., 1999). However, subsequent work with other inhibitors cast doubt on whether PKC isoforms are truly the relevant H89 target in this case (Lee and Linstedt, 2000).

More recently, contemporary MS phosphoproteomics has revealed new modes of COPII regulation. For example, the Luini group used engineered cell systems that experience a sudden surge in folded model COPII cargoes (e.g., VSVG, procollagen I) to examine phosphorylation changes occurring downstream (Subramanian et al., 2019). The authors reported the intriguing observation that Sec24, when engaged with folded cargo at ERES, appears to serve as a GEF for the heterotrimeric G protein Gα12, leading to Gα12 activation and subsequent adenylate cyclase 7 (ADCY7) and PKA signaling at ERES (Subramanian et al., 2019). These results suggest a new self-regulatory signaling circuit at the ER, where Sec24 senses cargo load by engaging directly with folded client proteins and then potentiates ER exit via a Gα12/ADCY7/PKA cascade (Subramanian et al., 2019). The authors dubbed this process “autoregulation of ER export,” or AREX (Subramanian et al., 2019). Phosphoproteomics results suggested cargo-induced changes in the MAPK and PI3K/Akt pathways, in addition to Gα12/ADCY7/PKA, and indeed targeted inhibition of ERK, Akt or PKA blocked AREX-driven trafficking of VSVG and procollagen model cargoes, though not that of a human growth hormone reporter, also a COPII client (Subramanian et al., 2019). In future work, it will be important to determine both the biophysical basis of Gα12 activation by Sec24 and the substrate(s) of the Gα12/ADCY7/PKA, MAPK, and PI3K/Akt pathways most directly relevant to AREX and COPII trafficking, especially since these pleiotropic signaling cascades undoubtedly have many indirect effects, beyond regulating anterograde secretion.

## Conclusion and Future Directions

A quarter-century of pathbreaking COPII research has revealed fascinating detail on the foundational aspects of this essential cell biological process. Despite this progress, we still have much to learn. For example, our understanding of ERES organization, the control of vesicle size and scission, and the dynamic regulation of COPII trafficking flux all remain incomplete (Barlowe, 2020). In particular, we believe that the continued study of post-transcriptional regulation will shed new light on how COPII trafficking is integrated into eukaryotic cell biology, both by responding rapidly to changes in upstream signals, nutrient cues or stress, and by relaying information from the ER to other organelles to maintain cellular homeostasis. We envision that recent advances in quantitative PTM characterization by MS, single-cell analytic methods, CRISPR-based functional genetic screens and new super-resolution optical and cryo-electron microscopy imaging modalities in particular will empower future studies of COPII regulation. Exciting advances on these fronts promise that the next 25 years of COPII research will vastly increase our understanding of this ancient and fundamental aspect of eukaryotic biology.

## Author Contributions

All authors participated in researching, writing, and revising the article and figures.

## Conflict of Interest

The authors declare that the research was conducted in the absence of any commercial or financial relationships that could be construed as a potential conflict of interest.
